# Bonding Without Bridging: Social Capital, Integration, and Well-Being Among Filipina Marriage Migrants in South Korea

**DOI:** 10.3390/ijerph23030305

**Published:** 2026-02-28

**Authors:** Asterio T. Miranda, Juneth Lourdes F. Miranda, Eungi Kim

**Affiliations:** 1 Department of Accounting and Taxation, College of Business, Keimyung University, Daegu 42601, Republic of Korea; 2 Lyceum of Cebu, Inc., Capitol Hills, Cebu City 6000, Philippines; 3 Department of Library and Information Science, Keimyung University, Daegu 42601, Republic of Korea

**Keywords:** bonding capital, bridging capital, ethnic enclaves, marriage migration, media discourse, social integration, South Korea

## Abstract

**Highlights:**

**Public health relevance—How does this work relate to a public health issue?**

- This study examines persistent social isolation among marriage migrants as a structural health-relevant condition.
- It analyzes how marriage-based migration pathways constrain everyday social participation.

**Public health significance—Why is this work of significance to public health?**

- Strong ethnic community participation coexists with sustained social isolation regardless of residence duration.
- A systematic gap exists between migrants’ lived experiences and public discourse representation.

**Public health implications—What are the key implications or messages for practitioners, policy makers, and researchers in public health?**

- Migrant-only support services may leave social isolation-related health vulnerabilities unaddressed.
- Integration strategies require sustained cross-group interaction opportunities alongside ethnic community centers.

**Abstract:**

This study examined whether strong ethnic community participation facilitates social integration or reinforces social separation among Filipina marriage migrants in the Daegu–Gyeongbuk region of South Korea. A mixed-methods design combined survey data collected between 2018 and 2019 with a media discourse analysis covering 2020 to 2025. Survey results indicate extensive ethnic network participation, with 94.5% of respondents involved in religious or Filipino community organizations, yet persistent integration challenges. Language barriers were reported by 54.8% of respondents and cultural misunderstandings by 40%, suggesting strong bonding social capital alongside limited bridging social capital even after prolonged residence. Drawing on Putnam’s social capital theory, 328 news articles on Filipino–Korean relations were screened, of which only 10 directly addressed marriage migrants. None examined the routine experiences identified in the survey, reflecting discursive erasure shaped by polarized narratives of victimization or exceptional success. The temporal separation between the datasets enables an assessment of whether documented integration patterns are acknowledged in public discourse. The findings raise concerns about policy approaches that prioritize ethnic community centers without providing sustained opportunities for intercultural interaction, particularly given that many respondents entered marriage through religious matching programs that embedded them within ethnic networks, with potential health implications.

## 1. Introduction

Filipina marriage migrants in South Korea often sustain dense ethnic and religious networks that provide emotional stability, cultural continuity, and practical support. In regions such as Daegu and Gyeongbuk, Filipino Catholic churches and community spaces function as key social infrastructures through which women maintain language use, social ties, and a sense of collective identity. These settings mitigate everyday marginalization in unfamiliar environments. However, participation in such networks does not necessarily translate into broader inclusion in Korean social life and may coexist with sustained social distance from the host society, with implications for migrant well-being given the centrality of social integration to psychological and long-term health outcomes.

Prior research indicates that high engagement in ethnic religious and community organizations often coexists with persistent barriers to social integration [1]. Language difficulties and limited friendships with host-society nationals remain common even among long-term residents, suggesting that duration of residence and functional adaptation to institutions do not ensure relational or affective integration. Drawing on Putnam’s distinction between bonding social capital within groups and bridging social capital across groups, this study conceptualizes this pattern as a bonding–bridging gap [2]. Ethnic institutions provide protection and practical resources, yet they rarely generate the cross-group ties required for sustained participation in mainstream social contexts.

International marriage migration in South Korea provides a critical context for examining integration dynamics among Filipina migrants. Since the 1990s, international marriage has expanded rapidly, especially between Korean men and women from Southeast Asia, shaped by rural demographic imbalance, gendered labor migration, and the institutionalization of transnational matchmaking through commercial and religious intermediaries [3,4]. According to Statistics Korea [5], marriages between Korean nationals and Filipino spouses numbered 422 in 2022, 388 in 2023, and 363 in 2024, showing a declining trend over the three-year period. In 2024, Filipino–Korean marriages ranked fifth among all source countries, alongside Cambodia, following China, Vietnam, the United States, and Japan. Filipina marriage migrants represent an important case for examining these dynamics, given the Philippines’ distinctive position in global migration systems [6]. Despite possessing resources often assumed to facilitate integration, they continue to face persistent challenges, raising fundamental questions about whether individual-level advantages can overcome structural constraints embedded in marriage-based migration pathways.

Recognizing these challenges, government responses have centered on the Multicultural Family Support Policy, which has provided language instruction, cultural orientation, and family counseling since 2008 [7]. Critical scholarship argues that these programs prioritize assimilation into dominant norms rather than reciprocal intercultural exchange and often limit migrant agency by emphasizing formal adjustment over relational integration [8,9]. Despite these policy efforts, important research gaps persist. First, it remains unclear whether ethnic bonding capital facilitates broader integration over time or functions as a stable alternative to host society engagement. Second, limited research systematically compares migrants’ lived experiences with their representation in public discourse, leaving unclear whether integration challenges are recognized in policy debates. Third, despite their distinctive characteristics, Filipina marriage migrants’ specific integration patterns have received relatively little focused empirical attention in South Korea.

Against this background, this study examines how strong ethnic networks coexist with persistent social isolation among Filipina marriage migrants and how this ordinary experience is represented in public discourse. Specifically, it addresses three questions: (1) What levels of adaptation do Filipina marriage migrants report across social, cultural, physical, and institutional domains? (2) How does media discourse frame the experiences of marriage migrants? (3) What do gaps between lived experience and media representation reveal about the nature and persistence of integration barriers? By juxtaposing research evidence on lived integration patterns with media framing under a policy regime oriented toward ethnic community infrastructures, this study identifies a structural misalignment among empirical realities, public discourse, and policy premises that constrains problem recognition and integration policy design. To address these questions, this study combines survey data with a systematic media discourse analysis.

This paper proceeds as follows: Section 2 reviews literature on social capital theory, marriage migration in South Korea, and media representation to establish the theoretical foundation. Section 3 presents the mixed-methods design combining survey data (2018–2019) and media analysis (2020–2025). Section 4 and Section 5 present results from each phase. Section 6 discusses structural persistence of integration barriers, media erasure patterns, and policy implications. Section 7 addresses study limitations. Section 8 concludes with implications for integration policy and future research.

## 2. Literature Review

Research on migrant integration emphasizes the interaction between social capital, migration pathways, and public representation. Studies consistently show that integration outcomes depend not only on individual characteristics but also on the structure of social networks, gendered migration regimes, and media discourse that shapes policy assumptions. This section reviews social capital theory, marriage migration in South Korea, and media representation to situate the study’s analytical framework and identify remaining gaps.

### 2.1. Theoretical Framework: Social Capital and Migration

The distinction between bonding and bridging social capital provides the theoretical foundation for this study [2]. Bonding capital refers to networks within ethnically homogeneous groups, such as co-ethnic friendships, ethnic churches, and community organizations. These networks offer emotional support, cultural familiarity, and practical assistance that facilitate everyday functioning [10]. At the same time, dense bonding networks can strengthen ethnic enclaves and reduce opportunities for sustained interaction with the host society.

Bridging capital refers to social ties that cross ethnic boundaries, including friendships with host nationals and participation in mainstream institutions [11]. Such ties facilitate integration by supporting language development, cultural competence, employment access, and information flows unavailable within ethnic enclaves [12]. Integration often requires both forms of capital, but their relationship is not sequential or automatic [13]. The central question concerns when bonding capital supports broader integration and when it substitutes for bridge building, reducing incentives or opportunities for cross-group engagement [10].

Barry [14] argued that multicultural policies emphasizing group-specific support may reinforce ethnic enclaves and bonding capital at the expense of common citizenship bonds. This critique addresses when cultural accommodation supports integration versus when it creates parallel social worlds with strong internal ties but limited mainstream engagement. Marriage migration intensifies this dilemma because household-based entry pathways constrain autonomous access to bridging opportunities from the outset.

Debates persist regarding whether ethnic enclaves facilitate or hinder integration. Classical assimilation theory viewed enclaves as delaying integration by reducing host society contact [15]. Later scholarship emphasized their role in providing essential resources that may enable longer term adaptation [16]. A key distinction is between enclaves as launching spaces that support eventual engagement and enclaves as stable environments that reduce the need for cross-cultural interaction [17,18]. Marriage migration may intensify this tension because household-based migration pathways constrain mobility and external social contact more strongly than labor or student migration.

### 2.2. Marriage Migration in South Korea

International marriage migration has become a significant demographic feature in East Asia. In South Korea, the phenomenon expanded in the 1990s as rural and working class men increasingly married women from other Asian countries due to gender imbalances and rural depopulation [3,4]. Filipina marriage migrants constitute a distinct subgroup characterized by English proficiency, Catholic religious backgrounds, and transnational networks shaped by the Philippines’ long history of labor migration [6,19]. Many entered Korea through the Unification Church’s international matching programs rather than commercial brokers, creating distinctive pre-migration conditions and post-arrival community structures [20].

Marriage migration in Korea is strongly gendered. Filipina spouses face unequal power relations within households and state institutions that structure their daily life, mobility, and civic participation [21]. Within this context, the patriarchal bargain framework explains how migrant women negotiate conformity to gender norms in exchange for family stability and legal security [3,22]. Unlike labor or student migrants who enter Korea via workplaces or educational institutions, marriage migrants enter via family relationships that often function as gatekeeping mechanisms, shaping access to language practice, social networks, and public participation [3]. Household-based migration constrains mobility and external social contact more strongly than employment or educational migration.

Kim and Vang [23] documented this dynamic in their study of Filipina marriage migrants’ voting practices, finding that women experienced political participation as a “family affair” shaped by gendered and ethnicized hierarchies. Despite legal citizenship, many women initially followed husbands’ or in-laws’ voting decisions due to perceived inferiority as “inauthentic” citizens. The study revealed that autonomous political decision-making emerged only when women developed economic independence, community engagement outside the family, and challenged their ascribed subordinate position—illustrating how household-based migration creates distinctive integration constraints that persist even after formal naturalization.

### 2.3. Media Representation and Integration Discourse

Media discourse plays a central role in shaping public understandings of migration and integration, influencing both policy design and societal attitudes [24]. Research on Korean media representations of marriage migrants identifies recurring patterns, including oscillation between victimization and celebratory narratives [25], emphasis on assimilation over multicultural inclusion [3], and reliance on exceptional success stories that obscure ordinary experiences [9]. Filipino marriage migrants are commonly portrayed through frames of cultural difference, family hardship, or occasional exceptional achievement [23].

These representations shape the boundaries of policy debate. When migration is framed primarily through individual success or failure, structural barriers and everyday forms of exclusion remain largely invisible [26]. Media coverage thus plays a constitutive role in determining which integration problems are recognized and which remain unaddressed. Examining the gap between media narratives and migrant experiences is therefore essential for assessing whether public discourse reflects integration realities or systematically distorts them.

### 2.4. Synthesis: Theoretical Gaps and Research Questions

Existing research establishes that bonding and bridging social capital function as distinct dimensions of integration, that marriage migration creates unique structural constraints, and that media representations shape public understanding of migrant incorporation. Three gaps remain. First, few studies empirically document whether high bonding capital within ethnic communities facilitates or substitutes for bridging capital over extended periods. Second, limited research systematically compares migrant self-reports with media analysis to assess representation gaps. Third, Filipina marriage migrants in Korea, despite their distinctive diaspora and religious networks, have received relatively limited focused analysis.

This study addresses these gaps through three contributions. First, it documents that strong ethnic community participation does not predict host society integration, even among residents with more than fifteen years of residence. Second, it employs temporal comparison, comparing survey data collected in 2018–2019 with media coverage from 2020–2025. This comparison demonstrates both the persistence of integration barriers and their systematic erasure in public discourse. Third, it identifies structural mechanisms that sustain the bonding and bridging gap in marriage migration contexts, moving beyond individual or cultural explanations to focus on institutional conditions that reproduce parallel social worlds.

## 3. Methodology

**Figure 1:**
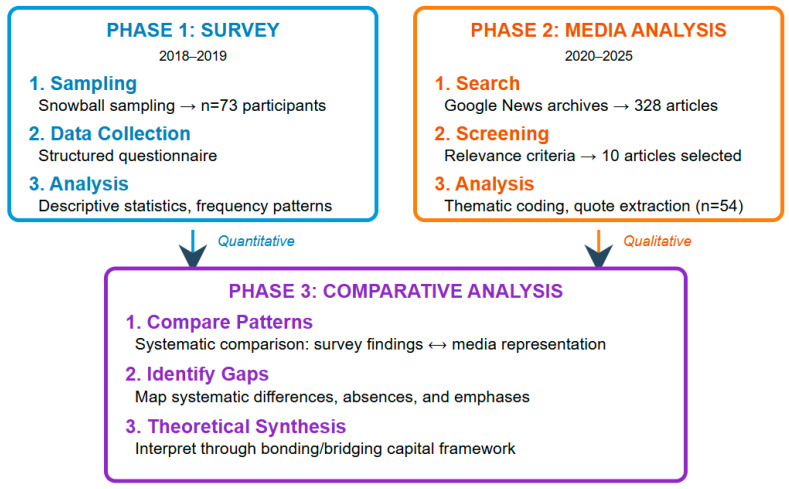
Mixed-methods research design integrating survey data (2018–2019) with media discourse analysis (2020–2025). Note. Each color represents a distinct research phase. → = sequential flow; ⟷ = bidirectional comparison.

This study employs a sequential mixed-methods design (Figure 1), integrating survey data collected in 2018–2019 with a content analysis of media coverage from 2020 to 2025. The temporal separation serves a theoretical purpose: testing whether the bonding–bridging gap represents a stable structural pattern or a transitional phase. If structural, it should persist and either appear or remain absent in subsequent media discourse. The design can demonstrate whether documented integration patterns find public representation; it cannot track individual changes or establish causal links between datasets. This constitutes a parallel analysis of experience versus representation, assessing structural persistence and representational gaps.

### 3.1. Phase 1: Survey Data Collection (2018–2019)

We recruited seventy-three Filipina marriage migrants in the Daegu–Gyeongbuk region between December 2018 and January 2019 using convenience sampling with snowball recruitment. Church leaders and multicultural center staff helped identify initial participants from their congregations and networks, who then referred other eligible Filipina marriage migrants. These gatekeepers also distributed anonymous surveys to eligible participants. This recruitment strategy likely introduced selection bias toward women who were already connected to religious or community organizations.

Participants completed self-administered anonymous surveys in Filipino (Tagalog). The instrument, adapted from established acculturation measures [27,28], employed two types of items: (1) Adaptation ratings: Respondents rated perceived difficulty across social, cultural, physical, and politico-legal domains using five-point Likert scales (1 = not difficult to 5 = extremely difficult), with responses analyzed via weighted means (Equation (1)). (2) Limiting factors: Respondents selected from a multiple-response checklist of barriers including language, conflicts or misunderstandings, cultural differences, family relationships, food, and responsibility or authority, with multiple responses permitted.

Responses were analyzed using weighted means to measure the central tendency of difficulty ratings. The weighted mean was computed as

(1) $$W_{m}=\frac{\sum_{i=1}^{n}f_{i}x_{i}}{\sum_{i=1}^{n}f_{i}},$$

where $W_{m}$ denotes the weighted mean, $f_{i}$ denotes the frequency (weight) associated with rating i, $x_{i}$ denotes the difficulty rating value (1–5), and n denotes the number of scale points. The use of weighted means for summarizing survey responses follows established survey methodology practice [29].

Mean scores were interpreted using a five-point scale:

1.00–1.79: no difficulty and very high adaptation;

1.80–2.59: slight difficulty and high adaptation;

2.60–3.39: moderate difficulty and moderate adaptation;

3.40–4.19: great difficulty and low adaptation;

4.20–5.00: extreme difficulty and very low adaptation.

Higher difficulty scores indicate lower levels of adaptation, whereas lower difficulty scores indicate higher levels of adaptation. Survey responses were analyzed using descriptive statistics and weighted means.

#### Ethics Statement

This study was conducted in accordance with the ethical principles of the Declaration of Helsinki. Pursuant to Article 15(2) of the Bioethics and Safety Act of the Republic of Korea (Act No. 18744) and Article 13 of its Enforcement Rule, research that involves only anonymous survey data from adult participants and does not collect identifiable or sensitive personal information is not subject to Institutional Review Board (IRB) review. As this study met these conditions, IRB review and approval were not required. Participation was voluntary, informed consent was provided in the questionnaire cover letter, no personal identifiers were collected, and all analyses were conducted using aggregated data only.

### 3.2. Phase 2: Media Content Analysis (2020–2025)

We conducted systematic searches of Google News archives spanning five years (2020–2025) to identify media articles addressing Filipino marriage migrants in South Korea.

#### 3.2.1. Search Strategy and Protocol

To ensure comprehensive coverage and maintain focus on marriage migration, we employed a structured Boolean search strategy using Google News archives. Ten focused search queries were constructed, each requiring the simultaneous presence of multiple key terms using AND logic (Table 1). All searches combined nationality identifiers (“Philippines,” “Filipino/a”), migration-related terms (“marriage,” “migrant,” “spouse,” “bride”), and geographical markers (“Korea/Korean”) to target content specifically ad-dressing Filipino marriage migrants rather than broader Filipino–Korean relations or other migration categories.

#### 3.2.2. Screening and Selection Process

Initial searches across ten Boolean query strings yielded 328 unique articles referencing Filipino–Korean relations. Articles were screened using three criteria: primary focus on marriage migration, explicit reference to Filipino or Filipina marriage migrants in South Korea, and substantive discussion beyond brief statistical mention. Items addressing Filipino–Korean relations more generally, other nationalities’ marriage migration, or duplicate records were excluded. This process yielded 10 articles, representing 3 percent of the initial results. The implications of this low yield are discussed in Section 5.1.

Table 2 lists the selected articles by source, date, and title. Six originated from Korean English-language outlets, including Korea Times and Korea.net, and four from Philippine sources, including The Philippine Star, GMA News, and ABS-CBN. Given the small corpus, qualitative thematic analysis was employed. All articles were coded by a single researcher using a predefined framework grounded in social capital theory (bonding and bridging), with consistent attention to framing patterns, voice allocation, and systematic absences. The analysis prioritized interpretive coherence over inter-coder reliability metrics due to the theoretically focused and limited sample size.

Furthermore, full-text extraction yielded fifty-four direct quotable passages across the ten articles. Each article was examined for dominant framing patterns, migrant portrayal, integration narratives, representation of ethnic networks, and voice and agency. Analysis focused not only on recurring themes but also on systematic absences, particularly the lack of coverage of ordinary migrants whose experiences were documented in the survey data.

### 3.3. Phase 3: Comparative Analysis

Following independent analyses of both datasets, we conducted a systematic comparison to identify convergences and divergences between lived experiences documented in survey responses and their representation in media discourse. Survey findings were mapped onto patterns of media coverage to examine whether integration challenges reported by respondents, including language barriers, social isolation, and reliance on ethnic networks, appeared in media narratives and how they were framed. Systematic gaps were identified by noting experiences that were widespread in the survey data but absent from media coverage.

This comparative approach enabled assessment of both the persistence of integration barriers and the role of public discourse in shaping policy responses to these barriers. Findings were interpreted through the bonding and bridging social capital framework to analyze how media representations either reveal or obscure the structural mechanisms sustaining the integration gap. The datasets address distinct analytical questions—migrant experiences (2018–2019) versus media representation patterns (2020–2025)—rather than tracking temporal change in the same population. The datasets span from 2018 to 2025, allowing assessment of whether 2018–2019 patterns appear in 2020–2025 media discourse.

## 4. Results: Survey (2018–2019)

### 4.1. Strong Community Networks and Persistent Integration Barriers

Despite extensive ethnic networks, persistent integration barriers remained (Table 3). Language barriers ranked first among factors hindering adaptation, cited by 40 respondents (54.8%). Two cultural challenges tied for second. Conflicts and misunderstandings were reported by 29 respondents (39.7%), as was difficulty understanding cultural differences (29 respondents, 39.7%). Family relationships ranked fourth, cited by 22 respondents (30.1%). These findings are notable given respondents’ long durations of residence. Twenty-one women arrived between 1996 and 2000, corresponding to more than twenty years of residence. Sixteen arrived between 2001 and 2005, corresponding to fifteen to twenty years of residence. Length of residence alone did not resolve language barriers or generate deeper cultural understanding.

### 4.2. Religious Affiliation and Organizational Membership

Survey data revealed substantial ethnic community infrastructure (Table 4). Sixty-nine of the 73 respondents (94.5%) reported religious affiliation, including 55 Catholics (75%) and 14 members of the Unification Church (19%). Only two respondents (3%) reported no distinct religious affiliation. Spouse religious affiliation differed substantially: among the 69 spouses who reported religious affiliation, 30 (43%) reported no distinct religion, 18 (26%) were Catholic, 15 (22%) were Buddhist, and 6 (9%) belonged to the Unification Church. This disparity indicates that religious networks primarily connect respondents to co-ethnic communities rather than to Korean society through shared religious practice with spouses.

Among those specifying organizational membership (*n* = 25), 13 belonged to the Family Federation for World Peace and Unification (52%) and 7 to the Daegu Filipino Catholic Center (28%). Additional memberships included Christian Church Organization (12%), Ilonggo Association in Korea (4%), and Network of Married Foreigners in Korea (4%). These patterns indicate the centrality of religious and co-ethnic infrastructures in providing social support and community connection.

Religious networks structured many women’s social worlds from the outset of migration, embedding them in dense co-ethnic ties. The prevalence of organizational membership in specifically Filipino or Filipino–Korean institutions, combined with the religious disparity between respondents and spouses, suggests that these networks function primarily as bonding capital within the ethnic community rather than as bridges to Korean society. This pattern corresponds to the strong bonding social capital observed throughout the study, including regular church attendance, Filipino friendships, and mutual aid networks.

### 4.3. The Bonding–Bridging Gap: Functional Competence Without Social Belonging

Analysis across adaptation dimensions reveals a patterned contrast consistent with the bonding–bridging gap (Table 5). Physical adaptation exhibited the lowest difficulty scores, indicating relatively high functional competence in everyday activities. Respondents reported comparatively little difficulty using transportation (mean = 2.07), shopping at markets (2.38), and accessing restaurants (2.49). These results suggest that most women were able to navigate public spaces and basic services in Korea with a moderate degree of ease, reflecting successful instrumental adjustment to daily life.

By contrast, social adaptation exhibited the highest difficulty scores among the measured domains, though the specific challenges varied across interactional dimensions. Reported difficulty was highest for dealing with unpleasant or aggressive behaviors (M = 3.38), followed by understanding humor (M = 3.04), making oneself understood in interactions (M = 2.99), and forming friendships with Korean nationals (M = 2.93). The mean difficulty score for social adaptation (M = 3.00) lies at the upper boundary of the moderate difficulty range (2.60–3.39) and approaches the threshold for great difficulty (≥3.40). These patterns indicate comparatively greater challenges in relational and communicative contexts than in other adaptation domains, with distinct interactional processes exhibiting different levels of difficulty. Taken together, the findings suggest functional competence in routine activities alongside persistent barriers in specific domains of social interaction, particularly those involving sustained interpersonal engagement with Korean nationals.

Table 6 presents respondents’ self-assessed levels of social adaptation. Overall, Filipina marriage migrants reported a moderate level of social adaptation, with a grand mean of 3.01 (SD = 1.10). The highest rated aspects were managing interactions with Korean nationals who display unpleasant or aggressive behaviors (M = 3.38) and relating to members of the opposite sex in Korea (M = 3.18). Moderate levels were reported for talking about oneself with Korean nationals and understanding Korean jokes and humor (both M = 3.04), as well as making oneself understood in conversations with Korean interlocutors (M = 2.99). Lower ratings were observed for relating to older Korean people and making friends with Korean nationals (both M = 2.93), attending social events and gatherings hosted by Korean nationals (M = 2.79), and living independently from family members overseas (M = 2.78). Variability was lower for the more concrete challenge of independent living (SD = 0.91) and higher for relational and cultural items such as understanding humor and making friends (SD = 1.21 and 1.20, respectively), indicating greater heterogeneity in respondents’ experiences of interpersonal integration.

Demographic characteristics condition opportunities for bonding and bridging (Table 7). Respondents’ mean age was 39.4 years (SD = 9.3) and husbands’ mean age was 47.9 years (SD = 7.2). Twenty-one of the 70 husbands with recorded age (30%) were in the 51–55 age bracket, whereas wives’ modal age bracket was 31–40. The mean spousal age gap was 8.5 years (SD = 6.3, range = 0–26 years). Although the average gap is moderate, the range extended to 26 years, indicating substantial variation in spousal age differences. Marriage pathways also structured social positioning. Among the 35 women (48%) who met their spouses through Unification Church mass blessing ceremonies, pre-marital communication was often limited, embedding respondents in religiously mediated networks at entry. Employment patterns further constrained opportunities for routine cross-group interaction. Of the 35 employed women, 15 worked in factories (43%) and 10 as housekeepers (29%). Twenty-two respondents (63%) worked part-time rather than full-time, limiting time availability and mobility for sustained interaction with Korean nationals.

## 5. Results: Media Analysis (2020–2025)

### 5.1. Media Scarcity and Limited Coverage

Systematic searches of Google News archives from 2020 to 2025 yielded striking results. Across 328 unique articles mentioning Filipino–Korean relations, only 10 (3%) specifically addressed Filipino marriage migrants. This scarcity confirms our central finding: ordinary migrants documented in survey data remain systematically absent from public discourse. The search process reveals a structured silence. Broad searches for “Filipino Korea” returned hundreds of articles covering diplomatic relations, overseas Filipino workers, and cultural events, whereas focused Boolean searches requiring all terms, namely “Philippines” AND “marriage” AND “Korean” AND “migrant,” reduced the yield to near zero. Even expanded five-year coverage produced minimal results, suggesting sustained rather than temporary invisibility. Whether this absence results from deliberate editorial choices, news values favoring exceptional cases, or structural constraints in news production cannot be determined; however, the pattern is consequential: marriage migrants are newsworthy only when they conform to specific narratives.

Notably, none of the ten articles addressed health dimensions of integration. Coverage focused on social, cultural, and economic aspects but did not examine migrants’ physical or mental health outcomes, healthcare access, or health-related challenges. This absence is significant, given that health represents a key domain of migrant well-being and integration.

### 5.2. Media Coverage Distribution by Frame

Qualitative analysis of ten articles, from which fifty-four direct quotes were extracted, revealed polarized coverage as shown in Table 8. Media frames were distributed as follows: six articles (60%) showcased exceptional success stories, including athletes, award winners, and celebrities who achieved public prominence. Two articles (20%) did not specify a clear integration frame, focusing instead on demographic statistics or general cultural content. One article (10%) portrayed migrants as victims of systematic disadvantage requiring institutional intervention. Only one article (10%) employed the “functions but isolated” frame, portraying migrants who function adequately while remaining socially isolated from Korean society—precisely the dominant pattern documented in the survey data (*n* = 73). This near-complete absence of ordinary migrant experiences may be consequential. Policy debates risk proceeding without acknowledging the lived reality of most marriage migrants, who maintain strong ethnic networks yet continue to face persistent barriers to social integration in Korean society.

### 5.3. Media Coverage Patterns: Success Stories and Victim Narratives

Success stories centered on named individuals whose achievements positioned them as model minorities. *Korea.net* profiled Byeon Jae-young, a Filipino–Korean athlete representing Korea internationally: “When I won for the first time wearing the national team uniform, I couldn’t express my joy in words” [36]. This framing emphasizes complete national belonging through achievement through wearing Korea’s uniform and representing the nation. Celebrity coverage followed similar patterns. ABS-CBN featured Kristel Fulgar teaching her Korean husband to cook adobo, presenting integration as charming cultural exchange rather than navigating structural barriers [34]. The Korea Times celebrated Global Korea Awards winners as second-generation Filipino–Koreans who “transformed challenges into resilience” and were “excelling in academics, athletics, arts, and community leadership” [38].

These success stories share key features: exceptional individual achievement, institutional validation (citizenship, awards, national team selection), and independence from ethnic community support. Integration appears as transcending the ethnic enclave rather than functioning within it. Notably absent: any mention of reliance on Filipino networks, the very support system 94.5% of our survey respondents utilized. In media narratives, successful integration means leaving the community behind, not depending on it.

Victim narratives portrayed migrants as passive recipients of structural violence. The Korea Times reported that foreign women who migrated to Korea to marry Korean men demonstrated a more than twofold chance of experiencing depression [31]. The article emphasized deteriorating rather than improving conditions. Among respondents with limited Korean language skills, 31.8% experienced depression, compared to 23.2% of those with advanced Korean abilities [31]. Language appears not as a manageable challenge but as a determining factor in mental health outcomes. Longer residence correlates with worse outcomes, suggesting systemic failure rather than gradual adaptation.

The gap between media coverage and survey reality has consequences. Public discourse oscillates between celebrating exceptional achievers and rescuing vulnerable victims, erasing the experience of ordinary migrants who function competently through ethnic networks and remain socially isolated. Policy debates proceed without acknowledging this majority. Integration measures target either exceptional achievement or crisis prevention, leaving unaddressed the sustained social isolation experienced by migrants like our survey respondents. These women shop, work, raise families, and attend church regularly, yet most cannot form friendships with Korean nationals or feel socially included. Media invisibility allows ineffective policies to persist.

### 5.4. Community Infrastructure and Limited Media Coverage

Although 94.5% of survey respondents reported regular participation in Filipino community-based support systems that structured their everyday social lives, media coverage largely ignored these connections. Seven of the ten articles made no reference to migrants’ ongoing community ties, and those that did mentioned them only instrumentally. When Danuri services were discussed, Lee Sabel described the organization’s role as providing “assistance in our community regarding work or jobs and the connection between the person and the community” [30]. Such references positioned community ties as channels for service delivery rather than as primary sites of belonging and social organization.

Success-oriented narratives were particularly likely to erase sustained community reliance. The *Korea.net* honorary reporter article on Seollal emphasized acceptance within Korean families rather than participation in Filipino community life [39]. Similarly, the *Philippine Star* profile of a reverse migration success story framed the Korean husband’s engagement with Filipino culture as a product of marital intimacy rather than involvement in migrant communities [32]. In these accounts, community connections appeared, if at all, as temporary stepping stones to be left behind rather than as enduring support structures.

This framing reflects a fundamental misrecognition of how ordinary migrants manage integration. Survey data showed that community-based ties provided practical assistance such as childcare, employment information, and language support, alongside emotional stability and cultural continuity. Media erasure of these structures reflects an implicit assumption that successful integration requires independence from migrant communities. This assumption is contradicted by the sustained reliance on Filipino social ties among respondents. Notably, over half had resided in South Korea for more than 10 years (Table 7), demonstrating that ethnic network reliance persists over time.

### 5.5. Voice and Authority in Media Coverage

Analysis of voice allocation reveals clear hierarchies of authority. In success-oriented narratives, migrants’ voices were more prominently featured, albeit through media mediation. For example, Korea.net quoted Byeon Jae-young describing his emotional experience representing Korea [36], and Philippine Star quoted Mary Buenaventura articulating her professional philosophy: “I love my profession. I am passionate about leading the business with a client-first and people-first approach. However, my husband and my son are the whys in my life that drive me to be passionate in what I do” [32]. In these cases, migrants were afforded space to narrate achievement in their own words within celebratory media frames.

In contrast, victim-oriented and policy focused coverage centered expert and official voices rather than migrant testimony. A Korea Times article on depression quoted researchers discussing correlations and patterns without including direct accounts from migrants experiencing these challenges. Policy articles relied on service providers and officials describing programs and administrative mechanisms, positioning migrants as populations to be assisted rather than as knowledgeable actors. Ordinary migrants who were neither exceptionally successful nor in crisis were entirely absent. Their everyday lives and integration efforts were treated as insufficiently newsworthy, leaving the modal experience documented in the survey without representation in media discourse.

### 5.6. Media Representation and the Bonding–Bridging Gap

Korean sources, including the Korea Times and Korea.net, accounted for 60% of the coverage and primarily presented success narratives and policy-oriented discussions emphasizing South Korea’s multicultural infrastructure and migrant achievement within Korean institutional contexts. Philippine sources, including The Philippine Star, GMA News, and ABS CBN, comprised 40% of the coverage and displayed greater variation, including reverse migration success stories, depersonalized statistical reporting, and celebrity focused entertainment cases. Neither source type systematically addressed everyday migrant experiences. Media coverage therefore functions to reproduce rather than critically examine the bonding–bridging gap. By emphasizing individuals who transcend ethnic communities through notable success or those requiring institutional intervention through victim-oriented narratives, media representations obscure the central survey pattern of migrants who function adequately within ethnic enclaves yet remain socially isolated from broader Korean society.

This polarization serves clear ideological functions. Success narratives tend to validate multicultural policies by portraying integration barriers as surmountable through individual effort, whereas victim narratives operate to legitimize paternalistic interventions by framing migrants as dependent populations. Absent from both frames is the dominant reality of migrants who neither achieve exceptional visibility nor experience acute crisis, who rely on ethnic networks without professional mediation, and who manage integration barriers without dramatic breakdown. As a result, policy debates proceed without engagement with the functioning but socially isolated majority.

The divergence between survey findings and media portrayals is substantial, as shown in Table 9. Integration measures, as reflected in media discourse, concentrate on either exceptional achievement, such as language competitions and cultural ambassador programs, or crisis prevention, including domestic violence hotlines and mental health screening. The persistent social isolation experienced by ordinary migrants is evident across the majority of survey respondents based on multiple adaptation indicators. This experience is absent from media coverage and remains unaddressed.

## 6. Discussion

This study identifies a persistent integration pattern among Filipino marriage migrants in South Korea. Strong ethnic community participation coexists with sustained social isolation from Korean society. Survey data show that 94.5% of respondents participated in religious communities, yet 54.8% reported ongoing language barriers and most reported difficulty forming Korean friendships despite long-term residence. Media coverage largely erased this ordinary experience by alternating between exceptional success narratives and victim-focused accounts. This discussion addresses three implications. First, it explains why integration barriers persist over time. Second, it examines how media erasure sustains ineffective policy responses. Third, it analyzes why bonding social capital does not translate into bridging social capital in marriage migration contexts and considers implications for policy and theory.

### 6.1. Structural Persistence of Integration Barriers

The comparison between survey data from 2018–2019 and media coverage from 2020–2025 indicates that the integration pattern documented in the survey—namely, functional competence alongside social isolation—remains absent from public discourse. Although individual trajectories cannot be tracked, the continued absence of this pattern in recent media coverage suggests that it remains marginal in public debate and problem recognition and may indirectly shape policy priorities. This analysis does not assume a deterministic pathway from media coverage to policy development. Respondents surveyed in 2018–2019 reported high levels of ethnic network participation alongside persistent language and cultural barriers. Contemporary media accounts describe similar difficulties, indicating limited substantive change over time.

Notably, long-term residents with more than twenty years of residence in Korea reported integration challenges comparable to those of more recent arrivals. This finding directly contradicts time-based assimilation models that predict gradual convergence through prolonged residence [15]. Instead, integration appears to depend on access to structured intercultural opportunities rather than length of stay, consistent with prior European research [11]. The continued presence of these barriers, despite evolving multicultural policy discourse, points to structural constraints that cannot be resolved through individual adaptation or the passage of time alone.

### 6.2. Limited Media Coverage of Ordinary Migrants

Comparative analysis reveals a clear gap between lived experience and media representation. Survey respondents managed everyday life competently by shopping, using transportation, and accessing services, yet remained socially isolated from Korean society. They possessed substantial bonding capital through religious and ethnic networks but lacked bridging ties to Korean nationals. Media coverage largely erased this modal experience through polarized framing. Half of the articles focused on exceptional achievers such as celebrities and athletes, one fifth on victimization involving abuse or discrimination, and the remaining 30% on celebrity human interest or policy services, with none addressing ordinary functioning migrants. Both success and victim frames describe real cases, but neither reflects the dominant reality of ordinary migrants who function without crisis or distinction yet remain excluded from Korean social life.

This pattern serves ideological functions. Success narratives validate assimilationist assumptions by implying that barriers can be overcome through individual effort. Victim narratives legitimize intervention programs by positioning migrants as dependent populations. Neither frame captures the bonding–bridging gap identified in the survey. As a result, public discourse fails to recognize the population most affected, potentially allowing ineffective integration policies to persist. The media search process suggests limited visibility. Even when searching specifically for marriage migration content across five years (2020–2025), only 10 articles emerged from an initial pool of 328 articles on Filipino–Korean relations. Our search strategy prioritized precision and may have missed some coverage, as discussed in Section 7. Nevertheless, this low yield indicates that marriage migrants receive minimal dedicated coverage. They are framed neither as temporary labor nor as fully integrated citizens. This pattern is consistent with structural invisibility in public discourse.

### 6.3. Bonding Without Bridging and Its Implications

Survey respondents demonstrated high levels of bonding social capital through religious affiliation, organizational participation, and mutual aid networks [10,13]. These networks provided emotional support and practical assistance but did not generate bridging capital to Korean society. The reason is structural rather than cultural. Bonding networks connect similar individuals, whereas bridging capital requires sustained cross-group interaction. Filipino religious communities offer support but few opportunities for regular social contact with Korean nationals.

Media discussions frequently referenced language barriers but framed them as solvable through government programs or individual study. This framing ignores the structural requirement for informal interaction with patient and invested native speakers. Government classes provide functional literacy but cannot substitute for conversational competence developed through everyday social relationships. Infrastructure that could foster bridging capital, such as integrated workplaces, mixed-neighborhood activities, and institutions that create weak ties [12], remains limited. Marriage migration further constrains access to such spaces through gendered household hierarchies that regulate time, mobility, and social contact [3,22]. The way couples met intensifies this constraint. Forty-eight percent married through religious mass blessings with minimal premarital communication, embedding them within religious enclaves upon arrival and making ethnic communities their primary social infrastructure rather than a transitional support.

This pattern mirrors Kim and Vang’s [23] finding that marriage migrants who transitioned to independent voting did so not merely through duration of residence but through specific enabling factors: economic autonomy, leadership roles in co-ethnic organizations, and development of collective identity as “multicultural family” members rather than subordinate family dependents. Their concept of “cultural citizenship” provides theoretical grounding for understanding how the bonding–bridging gap persists: ethnic networks offer essential support but remain embedded within family structures that impose conformity expectations, limiting opportunities for autonomous engagement with Korean society. These findings have clear policy and theoretical implications. Current multicultural policies emphasize migrant-only services and ethnic community centers, which strengthen bonding capital but, in the absence of parallel bridging mechanisms, may reinforce parallel social worlds [8,9].

These findings align with Barry’s [14] critique that policies emphasizing ethnic infrastructure without cross-group interaction opportunities may sustain parallel social worlds rather than facilitate integration. Effective integration requires deliberate bridge-building mechanisms that bring migrants and Korean nationals into sustained and equitable interaction. Without such mechanisms, ethnic enclaves will continue to function as stable support structures. They will not serve as pathways to social inclusion. Theoretically, the findings are consistent with the view that bonding and bridging social capital operate as independent dimensions rather than sequential stages, challenging linear assimilation models and supporting multi-dimensional integration frameworks [2,13].

From a public health perspective, the bonding–bridging gap documented in this study may have important implications for migrant health trajectories [40]. While ethnic networks provide essential emotional support and cultural continuity, the absence of bridging capital could limit access to diverse health information sources, healthcare navigation assistance, and the broader social resources that facilitate health-promoting behaviors in host societies. The persistence of integration barriers among long-term residents suggests these patterns may become entrenched rather than resolved over time, pointing to structural health vulnerabilities that current integration policies may fail to address. The variability observed in social adaptation scores on relational items, however, suggests that health vulnerability linked to social isolation may not be evenly distributed within this population, warranting more targeted investigation.

## 7. Limitations

This study has several limitations. First, the survey sample (*n* = 73) was recruited through religious communities and Filipino organizations using convenience and snowball sampling. This strategy captured migrants with strong ethnic network participation but excluded more socially isolated individuals. As a result, the findings reflect integration patterns among migrants with high bonding capital and cannot be generalized to all Filipina marriage migrants in South Korea. The focus on the Daegu–Gyeongbuk region further limits generalizability.

Second, only 10 articles from 328 screened met the inclusion criteria. The 3% yield reflects genuine scarcity of coverage of marriage migrants, although the Google News search strategy prioritized precision over recall and may have missed articles using alternative terminology or embedded in broader discussions. Google News indexing may underrepresent smaller regional or Korean-language publications, and print-only or paywalled content could not be accessed. All coding was conducted by a single researcher without inter-coder reliability assessment. While a predefined framework (bonding/bridging capital, frame analysis) and direct quotations provide transparency, independent coding could have strengthened robustness.

Third, the temporal gap between data sources (2018–2019 survey, 2020–2025 media) demonstrates pattern persistence across time periods but does not track the same individuals longitudinally. The comparison shows structural persistence rather than individual trajectories. Thus, our approach examined parallel patterns across two data sources (lived experience vs. public representation) rather than employing triangulation to validate identical findings through multiple methods.

Fourth, the quantitative analysis is primarily descriptive. We report adaptation patterns and vulnerability indicators but do not test formal hypotheses or conduct multivariate analysis. This approach is appropriate given the small sample size, convenience sampling strategy, and the study’s conceptual rather than predictive aims.

Fifth, constructs of bonding and bridging capital were inferred from survey responses rather than directly measured through social network analysis. The study focused exclusively on Filipina marriage migrants, though integration patterns may differ for Filipino men married to Korean women or migrants of other nationalities. Future research should employ comparative and longitudinal designs with direct network measurement.

Lastly, the survey did not collect data on respondents’ health outcomes or healthcare experiences. The public health implications discussed in this paper are therefore theoretical inferences based on integration patterns rather than direct measurements of health status. Future research should incorporate health measures to empirically test whether the bonding–bridging gap correlates with measurable health disparities.

## 8. Conclusions

This study contributes a conceptual clarification of how strong bonding social capital can coexist with sustained social isolation among marriage migrants, rather than offering a causal explanation of integration outcomes. By juxtaposing migrant self-reports with media representations, the study identifies a structural mismatch between lived integration patterns and public discourse, sharpening social capital theory in the context of marriage migration. Even with high levels of participation in Filipino religious communities, many respondents continue to face language difficulties and report limited friendships with Korean nationals, including those who have lived in South Korea for many years. Media coverage from 2020 to 2025 largely overlooks this everyday reality, oscillating between success narratives and victimization frames and leaving little room for migrants who manage daily life within ethnic networks yet remain socially distant from the host society. These patterns have important public health implications. Social isolation, even among migrants who function adequately in daily life, represents a structural health risk that existing integration policies fail to address. The absence of bridging capital limits migrants’ access to health information, healthcare navigation support, and the social networks that facilitate health-promoting behaviors in host societies.

Two findings are particularly relevant for current integration policy. First, integration challenges do not diminish with time, calling into question assimilation models that assume gradual convergence through prolonged residence. Second, ethnic networks provide essential emotional and practical support but do not, on their own, generate cross-ethnic social ties. This is especially evident in cases where religious matchmaking, which accounted for 48 percent of marriages, embeds migrants in ethnic enclaves from the outset. Existing policies emphasize ethnic community centers and migrant-focused services, reinforcing bonding capital without creating conditions conducive to bridging. These findings suggest that integration depends less on time or individual effort and more on institutional opportunities for sustained interaction between migrants and host society members.

Effective integration requires deliberate bridge-building mechanisms that facilitate sustained intercultural contact as a multidirectional process involving both migrants and host society members [41]. Examples include workplace buddy systems pairing migrant and Korean employees, community programs co-led by both groups, and institutional funding tied to Korean participation in multicultural activities. These implications apply specifically to marriage migration contexts characterized by household-based entry pathways and dense ethnic community infrastructures, and should be interpreted within those structural conditions. Future research should examine whether the bonding–bridging gap documented here correlates with measurable health outcomes among long-term marriage migrants, and whether deliberate bridge-building interventions can improve both integration patterns and health indicators.

## Figures and Tables

**Table 1:** Google news Boolean search queries for media analysis.

Search Query	Boolean Logic
Philippines + marriage + Korean + migrant	Philippines AND marriage AND Korean AND migrant
Filipina + marriage + Korea + migrant	Filipina AND marriage AND Korea AND migrant
Filipino + wife + Korea + marriage	Filipino AND wife AND Korea AND marriage
Philippines + bride + Korea + marriage	Philippines AND bride AND Korea AND marriage
Filipino + spouse + Korea + migrant	Filipino AND spouse AND Korea AND migrant
Philippines + multicultural + Korea + marriage	Philippines AND multicultural AND Korea AND marriage
Filipino + multicultural + family + Korea	Filipino AND multicultural AND family AND Korea
Filipina + integration + Korea + marriage	Filipina AND integration AND Korea AND marriage
Filipino + migration + Korea + marriage	Filipino AND migration AND Korea AND marriage
Philippines + international + marriage + Korea	Philippines AND international AND marriage AND Korea

Note. All searches were conducted in Google News archives with date range filter: 1 January 2020–31 December 2025.

**Table 2:** Media articles analyzed in content analysis (2020–2025).

No.	Source	Type	Date	Article Title
1	Korea Times	Korean	21 February 2023	Multilingual Danuri helpline assists migrants in Korea [30]
2	Korea Times	Korean	1 January 2024	Migrant wives twice as likely to experience depression as Korean women [31]
3	Philippine Star	Philippine	14 February 2025	Real Korean-Filipino love story worthy of K-drama [32]
4	GMA News	Philippine	12 January 2021	Most Filipinos with foreign spouses are married to this nationality [33]
5	ABS-CBN	Philippine	11 August 2025	Kristel Fulgar teaches Korean husband how to cook adobo [34]
6	ABS-CBN News	Philippine	13 May 2025	Benedict Cua attends Kristel Fulgar’s wedding in South Korea [35]
7	Korea.net	Korean	6 March 2025	[Multicultural marvels] Korean Philippine world taekwondo champ [36]
8	Korea Times	Korean	14 November 2023	Korea Polytechnics expands vocational training for immigrants [37]
9	Korea Times	Korean	27 November 2025	Winners of 14th Global Korea Awards [38]
10	Korea.net	Korean	22 January 2020	How foreign wives of Koreans celebrate Lunar New Year [39]

Note. Korean sources: *n* = 6 (60%); Philippine sources: *n* = 4 (40%). All articles accessed through Google News archives and available online as of search date (2025).

**Table 3:** Limiting factors hindering adaptation (*n* = 73).

Limiting Factor	Frequency	Percentage	Rank
Language barriers	40	54.8%	1
Conflicts and misunderstandings	29	39.7%	2
Difficulty understanding cultural differences	29	39.7%	2
Family relationships	22	30.1%	4
Food preferences	16	21.9%	5
Responsibility and authority	11	15.1%	6

Note. Items with identical frequencies were assigned the same rank.

**Table 4:** Religious affiliation and organizational membership (*n* = 73).

Category	Characteristic	N	%
Respondent Religious Affiliation	Roman Catholic	55	75.34
	Unification Church	14	19.18
	No distinct religion	2	2.74
	Others (Tong-il)	2	2.74
	Total	73	100
Spouse Religious Affiliation *	Roman Catholic	18	26.09
	Buddhism	15	21.74
	Unification Church	6	8.7
	No distinct religion	30	43.48
	Total	69	100
Organizational Membership **	Family Federation for World Peace and Unification	13	52
	Daegu Filipino Catholic Center	7	28
	Christian Church Organization	3	12
	Ilongo Association in Korea	1	4
	Network of Married Foreigners in Korea	1	4
	Total	25	100

Note. * *n* = 69 as one respondent did not report spouse’s religious affiliation. ** Percentages calculated from 25 respondents who specified organizational membership.

**Table 5:** Adaptation levels by dimension.

Dimension	Weighted Mean
Physical Access	2.52 (High adaptation)
Politico-Legal	2.87 (Moderate adaptation)
Cultural	2.83 (Moderate adaptation)
Social	3.00 (Moderate adaptation)
Overall	2.805 (Moderate adaptation)

**Table 6:** Level of adaptation of Filipina marriage migrants along the social aspects.

Indicator	1	2	3	4	5	Mean	SD	Interpretation
Making friends with Korean nationals	10	9	31	12	11	2.93	1.2	Moderate
Making yourself understood when dealing or conversing with Korean nationals	10	9	30	18	6	2.99	1.12	Moderate
Talking about yourself with other Korean nationals	11	13	25	16	8	3.04	1.2	Moderate
Understanding jokes and humor the Korean way	12	12	23	19	7	3.04	1.21	Moderate
Dealing with Korean nationals having unpleasant or aggressive behaviors	16	12	30	14	1	3.38	1.07	Moderate
Relating to members of the opposite sex in Korea	9	14	34	13	3	3.18	1	Moderate
Living away from family members overseas/independently from your parents	3	10	33	22	5	2.78	0.91	Moderate
Relating to older or elder Korean people	7	15	22	24	5	2.93	1.09	Moderate
Going to social events, gatherings and other social functions hosted by Korean nationals	8	8	27	21	9	2.79	1.13	Moderate
Overall Mean Score						3.01	1.10	Moderate

Note. 1 = No Difficulty (Very high adaptation); 2 = Slightly Difficult (High adaptation); 3 = Moderately Difficult (Moderate adaptation); 4 = Greatly Difficult (Low adaptation); 5 = Extremely Difficult (Very low adaptation). SD = Standard Deviation.

**Table 7:** Demographic vulnerability indicators (*n* = 73).

Category	Characteristic	N	%
Wife Age	31–40 years	26	35.6
	41–50 years	24	32.9
	21–30 years	16	21.9
	Other ages	7	9.6
	Mean age	39.4 ± 9.3 years	-
	Total	73	100.0
Husband Age *	51–55 years	21	30.0
	46–50 years	18	25.7
	41–45 years	11	15.7
	Other ages	20	28.6
	Mean age	47.9 ± 7.2 years	-
	Total	70	100.0
Spousal Age Gap	Mean ± SD	8.5 ± 6.3 years	-
	Range	0–26 years	-
Marriage Pathway	Unification Church program	35	47.9
	Friend introduction	28	38.4
	Agency/broker	8	11.0
	Other	2	2.7
	Total	73	100.0
Employment Status	Employed	35	47.9
	Not employed	38	52.1
	Total	73	100.0
Employment Type **	Factory worker	15	42.8
	Housekeeper	10	28.6
	Other occupations	10	28.6
	Total	35	100.0
Work Schedule **	Part-time	22	62.9
	Full-time	13	37.1
	Total	35	100.0
Residence Duration	20+ years (1996–2000)	21	28.8
	15–20 years (2001–2005)	16	21.9
	10–15 years (2006–2010)	12	16.4
	Less than 10 years	24	32.9
	Total	73	100.0

Note. * *n* = 70 due to 3 widowed/separated respondents. ** Percentages calculated from 35 employed respondents.

**Table 8:** Media frame distribution (*n* = 10 articles, 2020–2025).

Frame	Description	*n*	%
Success story	Exceptional achievers overcoming barriers through individual effort or talent (athletes, award winners, celebrities)	6	60
Not specified	Articles without clear integration framing (demographic statistics, general cultural content)	2	20
Functions but isolated	Migrants managing daily life competently while remaining socially isolated from Korean society, with strong bonding capital but limited bridging ties	1	10
Struggling victim	Vulnerable populations facing systematic disadvantage requiring institutional intervention	1	10
TOTAL		10	100

**Table 9:** Survey vs. media comparison: the coverage gap.

Dimension	Survey (*n* = 73)	Media (*n* = 10)
Coverage of daily integration patterns	73 respondents documented	0 articles on daily integration experiences
Ethnic Networks	94.5% religious affiliation	70% not mentioned
Language Barriers	54.8% report	Only in victim frame
Integration Frame	Functions competently but socially isolated	60% exceptional success OR 10% victimization

## Data Availability

Anonymized survey data are not publicly available due to ethical considerations. Media sources used for analysis are publicly accessible and listed in the references.
